# Quality assessment of global health care system in the shadow of COVID-19: - a systematic review

**DOI:** 10.1186/s12889-023-15840-3

**Published:** 2023-05-26

**Authors:** Karuna Nidhi Kaur, Farah Niazi, Ruchi Thakur, Shazina Saeed, Shweta Rana, Harpreet Singh

**Affiliations:** 1 Laboratory of Disease Dynamics & Molecular Epidemiology, Amity Institute of Public Health, Amity University, Noida, India; 2 Amity Institute of Public Health, Amity University, Noida, India; 3 Division of Biomedical Informatics (BMI), Indian Council of Medical Research, Ansari Nagar, New Delhi, India

**Keywords:** Quality Assessment, Global Health, Covid-19, Health care system, Patient satisfaction

## Abstract

**Introduction:**

The healthcare system is critical to the country’s overall growth, which involves the healthy development of individuals, families, and society everywhere. This systematic review focuses on providing an overall assessment of the quality of healthcare delivery during COVID-19.

**Methodology:**

The literature search was conducted from March 2020 till April 2023 utilising the databases “PubMed,“ “Google Scholar,“ and “Embase.“ A total of nine articles were included. Descriptive statistics was performed using Microsoft Excel. PROSPERO registration ID- CRD42022356285.

**Results:**

According to the geographic location of the studies included, four studies were conducted in Asia [Malaysia(n = 1); India (Madhya Pradesh) (n = 1); Saudi Arabia(n = 1); Indonesia (Surabaya) (n = 1)], three in Europe [U.K. (n = 1); Poland (n = 1); Albania (n = 1)] and two in Africa [Ethiopia(n = 1); Tunisia (n = 1)]. Overall patient satisfaction was found highest among studies conducted in Saudi Arabia (98.1%) followed by India (Madhya Pradesh) (90.6%) and the U.K. (90%).

**Conclusion:**

This review concluded five different aspects of patients satisfaction level i.e. reliability, responsiveness, assurance, empathy, and tangibility. It was found that the empathy aspect had the greatest value of the five factors, i.e., 3.52 followed by Assurance with a value of 3.51.

## Introduction

Improving health is the main aim of every healthcare system. According to the World Health Organization (WHO), each national health system aims to achieve overall three goals which are good health, responsiveness to the expectation of people, and fairness to financial contribution. The objective of achieving good health includes attaining goodness along with health equity for every citizen. These goals could be successfully achieved only when each health system performs four vital functions that are quality service provision, resource generation, financing, and stewardship [1].

Progress towards achieving United Nations Millennial Development Goals or any national health program depends entirely on the health care system of that nation and the quality of care provided therein [1]. Quality of the health service is the assimilation of the health system environment, and actions of providers and individuals working within the system. To provide quality care, health services should be effective, safe, people-centered, timely, equitable, integrated, and efficient. Organizations such as WHO, Organization for Economic Co-operation and Development (OECD) and World Bank have proposed certain actions for key constituencies such as government, health system, citizens, patients, and healthcare workers that need to work together to accomplish the goal of quality of health services. The Sustainable Development Goals (SDG) 2015, are the WHOs shared plan that has emphasized on the quality of care as a key element of universal health coverage (UHC) which means all individuals and communities should receive health services without suffering from financial hardships [2]. UHC includes the full spectrum of essential, quality health services, from health promotion to prevention, treatment, rehabilitation, and palliative care across the life course. The countries that progress towards UHC will make progress towards other health-related targets as well, which will help to achieve good health, help people to earn and escape from poverty, and provide long-term economic development. This global crisis created by the COVID-19 pandemic could be an opportunity to make certain changes that benefit both UHC and health security [3].

The COVID-19 pandemic has posed a huge challenge for all healthcare systems and governments globally. In 2020 alone, there were an estimated 2.0 million lives lost to COVID-19. Moreover, indirect health loss due to lapses in the provision of essential health services such as vaccination, and emergency care were also major concern. The unprecedented situation has severely impacted the availability and the ability of the health system to provide undisrupted health services. In countries where health services were widely accessible and affordable, governments were finding it difficultto respond to the sudden growing needs of the population and increased cost of health services [4]. WHO and its partners have been helping countries to respond to the challenges that are being placed on their health care systems by strengthening primary healthcare and advancing universal health coverage [5]. It is essential to continuously monitor and evaluate the effectiveness of modifications done in the healthcare delivery system. In addition to the global pulse survey on the continuity of essential health services during COVID-19, other measures have also been proposed such as analyzing and using routine data for monitoring the effect of COVID-19 on essential health services. It is a practical recommendation on how to use health care indicators to analyze changes in the assessment and delivery of essential health services [6].

Service excellence revolves around three factors that is doctor, patient, and organization. Patient satisfaction is also one of the important and commonly used indicators to measure the quality of healthcare. It indicates how much a health care provider and health care institution are effective in rendering health services [7]. Measuring patient satisfaction helps to measure healthcare quality and thus helps in identifying ways to improve it. Patient satisfaction results from the patients’ understanding and acceptance of his or her health state, the quality of care and the extent to which care received has met their expectations. Pandemic situations like COVID-19 can affect the patient’s level of satisfaction. Both public and private health care institutions have assessed patient satisfaction based on different quality assessment components such as reliability, responsiveness, assurance, empathy, and tangibility. The service quality (SERVQUAL) technique is commonly used for evaluating the quality of service in a wide variety of service environments, sectors, and nations. Hospital Consumer Assessment of Healthcare Providers and Systems (HCAHPS), and other traditional patient satisfaction surveys are also used that may evoke a wide variety of answers from patients regarding the quality assessment of healthcare institutions [8]. This systematic review focuses on providing overall patient satisfaction concerning the quality assessment of healthcare delivery during the COVID-19 pandemic.

## Methodology

### Protocol Registration Methods

This systematic literature review followed the Preferred Reporting Items for Systematic literature reviews and Meta-Analyses (PRISMA) 2020 guideline and has been registered with the International Prospective Register of Systematic Reviews (PROSPERO) (Registration ID- CRD42022356285).

### Search strategy

PRISMA 2020 guidelines were used to design the methodology for this systematic review [9]. The PRISMA statement includes a 27-item checklist which assures transparency, iteration, and complete reporting for systematic reviews. The literature search was conducted from March 2020 till April 2023 using “PubMed”, “Google Scholar” and “Embase” databases with the combination of the following term sequences; - Patient Satisfaction, Quality Assessment of Health Care and COVID-19. A Manual search in web-based resources was accomplished on Google as illustrated in Table 1.

**Table 1:** Data Base Search

DATABASE SEARCH			
DATABASE	KEYWORDS	FILTER	RESULTS
PUBMED	“patient satisfaction“[MeSH Terms] OR patient satisfaction[Text Word] “COVID-19“[All Fields] OR “COVID-19“[MeSH Terms] OR “SARS-CoV-2“[All Fields] OR “sars-cov-2“[MeSH Terms] OR “Severe Acute Respiratory Syndrome Coronavirus 2“[All Fields] OR “NCOV“[All Fields] OR “2019 NCOV“[All Fields] OR ((“coronavirus“[MeSH Terms] OR “coronavirus“[All Fields] OR “COV“[All Fields])”Quality Assurance, Health Care“[Mesh] AND quality[All Fields] AND assessment[All Fields] “delivery of health care“[MeSH Terms] OR (“delivery“[All Fields] AND “health“[All Fields] AND “care“[All Fields]) OR “delivery of health care“[All Fields] OR (“healthcare“[All Fields] AND “delivery“[All Fields]) OR “healthcare delivery“[All Fields]	• Study Design: - Cross sectional studies. • Time Duration: - (2020–2023) • Language: - English.	1218
GOOGLE SCHOLAR	patient satisfaction, Quality Assessment of healthcare, COVID 19		23,800
EMBASE	TITLE-ABS-KEY (patient AND satisfaction, AND quality AND assessment, AND health AND care )		2347

The PICO was defined as follows:

Population (P)- COVID-19 survivors who were hospitalized.

Intervention (I)- Quality Assessment.

Comparison/Control group (C)- Pre-COVID era.

Outcome (O)- Quality assessment of the global health care system.

#### Selection criteria

The studies were selected on the basis of the inclusion and exclusion criteria as shown in Table 2.

**Table 2:** Inclusion and Exclusion Criteria

INCLUSION CRITERIA	EXCLUSION CRITERIA
Original articles evaluating the patient satisfaction level for the quality assessment of health care.	Studies not evaluating the patient’s satisfaction for the quality assessment of health care.
Only Cross-sectional study designs were included.	Studies focusing on specific disease like neurological disorder, ophthalmological disorder ,long COVID and specific department like ENT, Oncology department.
Studies published from March 2020 (advent of COVID-19) to April 2023.	Review articles, Case Report, Case Series, commentary, and correspondence were excluded.
Full Text articles published in English language.	Studies not published in English Language.

### Study selection

**Fig. 1:**
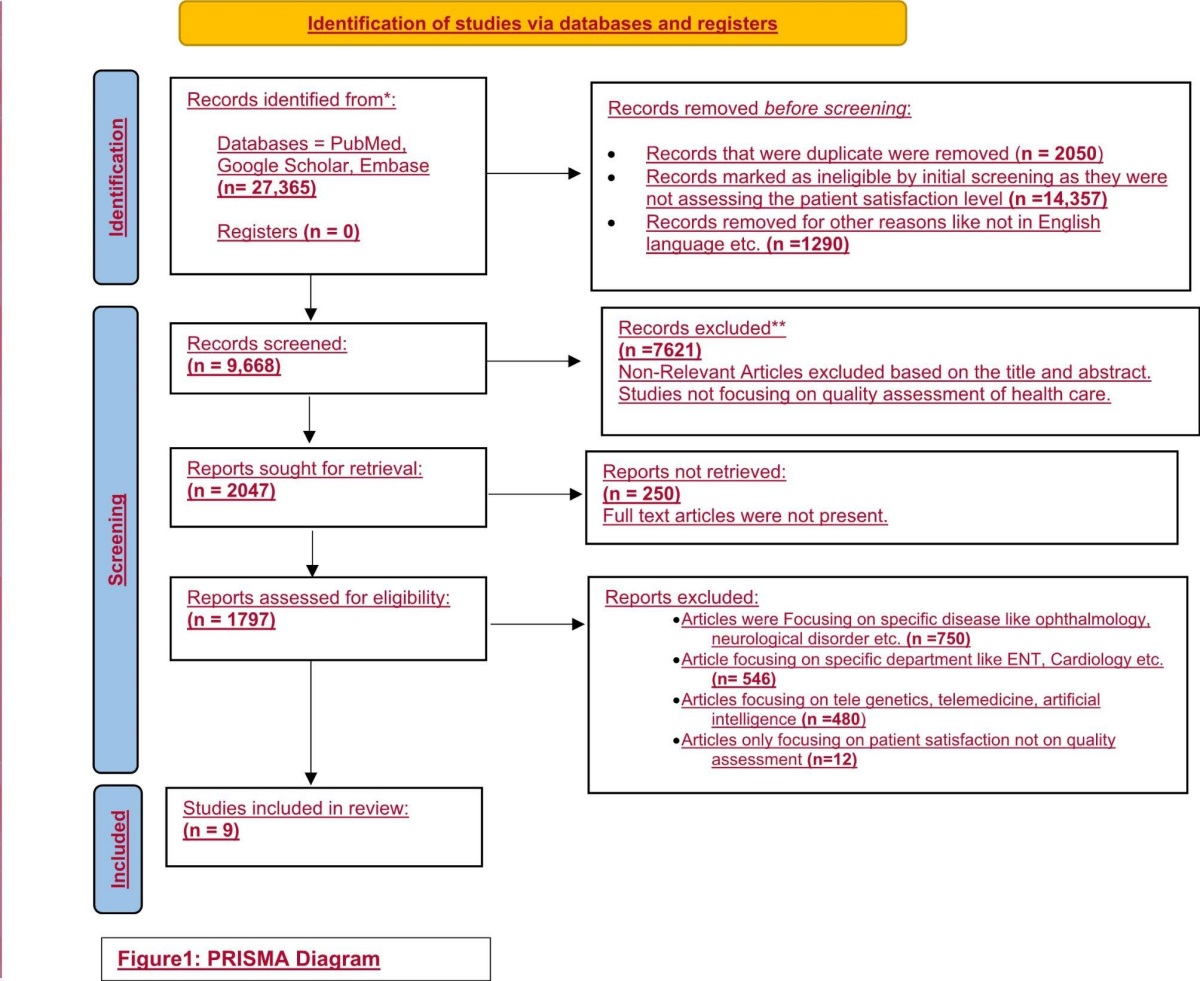
PRISMA Diagram

Two independent researchers selected the relevant studies in a two-step process. In the first step, we reviewed the title and abstracts of the studies for potential eligibility. For the second step, the full texts of these were obtained for in-depth evaluation. Only cross-sectional studies that had reported the patient satisfaction level for the quality assessment of health care were included. The initial data base search yielded 27,365 published articles matching the keywords over the last 3 years. After eliminating 2050 duplicate articles, 14,357 studies were excluded as they were not assessing the patient satisfaction level for quality of health. Furthermore, 1290 articles were also removed because of non-English publications or ineligible articles. The remaining 9668 articles were screened, and 7621 articles were excluded after the researcher read the abstract. For full text evaluation, 2047 articles were sought for retrieval of which 250 articles were not retrieved and 1797 articles were assessed for full text evaluation. Studies focusing on specific diseases like ophthalmology, neurological disorder, etc. **(n = 750)** or on specific departments like ENT, Cardiology, etc. **(n = 546)**, focusing on other aspects such as tele genetics, telemedicine, artificial intelligence **(n = 480**), articles only focusing on patient satisfaction and not on quality assessment **(n = 12)** were also excluded. After the final screening and evaluation, 9 articles that met the inclusion criteria were finally included as shown in Fig. 1.

### Data extraction

The data were extracted and collated into Microsoft Excel spreadsheets and the following domains of data were extracted from each study: study design, sampling techniques, study population, sample size, type of health care, type of scale used, etc. Studies were categorized based on service quality and patient’s satisfaction. Given the nature of the review question, a meta-analysis was not undertaken and instead, a descriptive synthesis and thematic analysis were done.

## Results

A total of nine studies were included in the analysis. An overview of each article was provided in Table 3, including details of the authorship, title of the study, year of the study conducted, study design, sampling technique, study population, age of participants, sample size, type of health care institution, questionnaire used, type of scale used and satisfaction percentage. According to the geographic location of the studies included, four studies were conducted in Asia [Malaysia(n = 1); India (Madhya Pradesh) (n = 1); Saudi Arabia(n = 1); Indonesia (Surabaya) (n = 1)], three in Europe [U.K. (n = 1); Poland (n = 1); Albania (n = 1)] and two in Africa [Ethiopia(n = 1); Tunisia (n = 1)]. The studies that were taken into consideration had different sample sizes, Poland (N = 98), India (Madhya Pradesh) (N = 150), Indonesia (Surabaya) (N = 200), Saudi Arabia (N = 223),Ethiopia (410), U.K.(N = 704), Albania (N = 800), Tunisia (N = 943)and Malaysia (N = 3025).

**Table 3:** Detailed Characteristics of studies included in the Review

S no.	Author Name	Title of the study	Year	Study design	Sampling technique	Study population	Age	Sample size	health care institution	Country/state/city	Questionnaire consists of:	Type of scale used	Satisfaction (%)
1	Laura Wulandari [18]	Customer satisfaction during covid-19 pandemic period at private clinic X Surabaya	2021	Cross-sectional study	Non-probability technique: Incidental technique	Patients admitted in clinic X	Not mentioned	200	Private clinic X	Indonesia (Surabaya)	Reliability, responsiveness, assurance, empathy, tangible	4-point Likert scale.	71.8
2	Thamer A. Bin Traiki et al. [10]	Impact of covid-19 pandemic on patient satisfaction and surgical outcome: A retrospective cross-sectional study	2020	Retrospective and cross-sectional study	Not mentioned	Patient admitted in King Khalid university hospital	Median age 53	223	university hospital	Saudi Arabia	Hospital Consumer Assessment of Healthcare Providers and Systems (HCAHPS) questionnaire	Not mentioned	98.1
3	Thomas Key et al. [11]	The Patient Experience of Inpatient Care During the COVID-19 Pandemic: Exploring Patient Perceptions, Communication, and Quality of Care at a University Teaching Hospital in the United Kingdom	2021	Cross-sectional study	Not mentioned	Hospital inpatients’	18–80+	704	Cardiff and Vale university Health Board	U.K.	Mixed method questionnaire	5-Point Likert scale	90
4	Berhanu Senbeta Deriba et al. [7]	Patient Satisfaction and Associated Factors During COVID-19 Pandemic in North Shoa Health Care Facilities	2020	Cross-sectional study	Systematic random sampling technique	Chronic disease patients: (HIV/AIDS, tuberculosis, cardiovascular disease, cancer, diabetes mellitus, chronic respiratory disease, and chronic musculoskeletal disease)	15->45	410	North shoa public health facility	Ethiopia (Oromia state)	Face to face interview by semi structured questionnaire	5-point Likert scale	44.6
5	Arvind Sharma et al. [12]	Satisfaction among COVID-19 Positive Patients: A Study in a Tertiary Care Hospital in Central India	2021	Retrospective cross-sectional study	simple random sampling	Covid-19 patients	18->60	150	Tertiary hospital	India (Jabalpur, Madhya Pradesh)	interview	Not mentioned	90.6
6	Faten Methammem et al. [15]	Patients’ Satisfaction with the Quality of Care in the Tunisian Private Hospitals during the Second Wave of COVID-19 Pandemic: Does Human Resource Planning Matter?	2022	Cross-sectional study	Not mentioned	Private hospital directors (n = 85) and private hospital patients (n = 858)	Not mentioned	943	Private Hospitals	Tunisia	self-administered questionnaire consisting of close-ended questions	7-point Likert scale	59.1
7	Afiq Izzudin A. Rahim et al. [8]	Patient Satisfaction and Hospital Quality of Care Evaluation in Malaysia Using SERVQUAL and Facebook	2021	Cross-sectional study	Universal sampling	Face book data (n = 1825), SERVQUAL data (n = 1200)	Not mentioned	3025	Government hospital	Malaysia	Utilized Facebook data and SERVQUAL data. SERVQUAL data includes Tangible, Reliability, Responsiveness, Assurance, Empathy	Not Mentioned	54.5
8	Magdalena Kludacz-Alessandri et al. [13]	The Quality of Medical Care in the Conditions of the COVID-19 Pandemic, with Particular Emphasis on the Access to Primary Healthcare and the Effectiveness of Treatment in Poland	2021	Cross-sectional study	Not mentioned	Patients of the Corten Medic primary healthcare facilities	< 25->65	98	Four primary health facility	Poland	Structured questionnaire	5-point Likert scale	71.1
9	Rezarta Kalaja et al. [14]	Patient satisfaction with quality of care in public hospitals in Albania	2022	Cross-sectional study	Simple random Sampling	Patients from ten selected hospitals	18 > 65	800	Public hospitals	Albania	SERVQUAL instrument	5-point Likert scale	67

Three out of nine studies, that were conducted in Indonesia (Surabaya), Malaysia and Albania used the SERVQUAL tool to assess the quality of care and patient’s satisfaction level. This tool focusses on assessing five components: reliability, responsiveness, tangibles, assurance, and empathy. Another study conducted in Saudi Arabia used the Hospital Consumer Assessment of Health Care Providers and System (HCAHPS) questionnaire, which was the first national, standardized, publicly reported survey for assessing the patient’s perception of hospitalcare and the remaining five studies used different collection tools such as mixed method questionnaire, interviews, and self-structured questionnaires. The studies were conducted in different types of institutions, with two studies in private hospitals/clinics, one in a tertiary hospital and six in public hospitals.

**Fig. 2:**
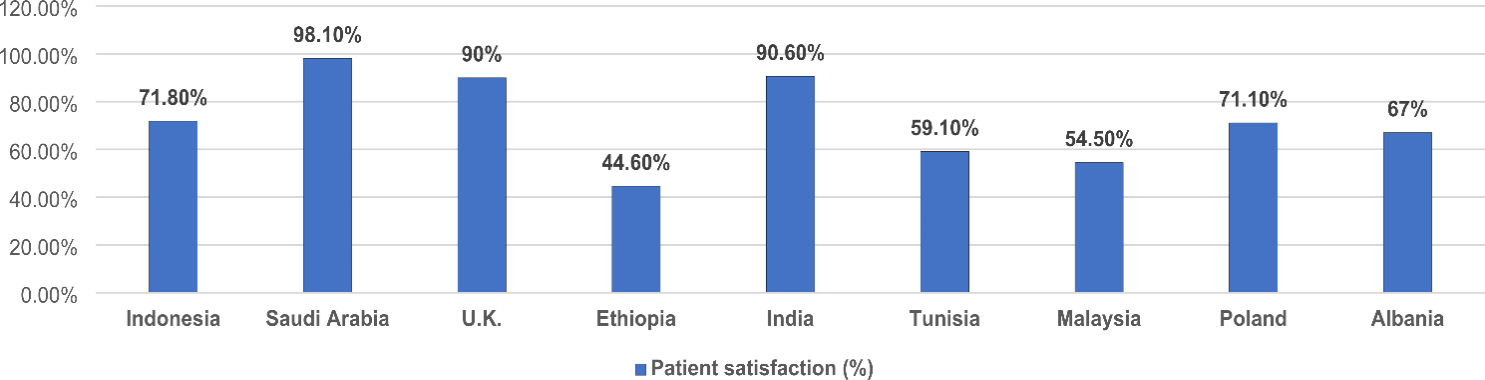
Overall Patient Satisfaction

Overall patient satisfaction was found highest amongst studies conducted in Saudi Arabia (98.1%) followed by India (Madhya Pradesh) (90.6%), U.K. (90%), Indonesia (71.8%), Poland (71.1%), Albania (67%), Tunisia (59.1%), Malaysia (54.5%) and Ethiopia (44.6%) as depicted in Fig. 2.

## Discussion

The nine studies collectively suggest that patient satisfaction during the COVID-19 pandemic varies depending on factors such as quality of care, communication, safety measures, and availability of personal protective equipment (PPE). Despite the challenges posed by the pandemic, overall satisfaction levels remained high in most cases. The review found that patient satisfaction varied across countries, with Saudi Arabia, India, and the U.K. reporting the highest levels of satisfaction, while Ethiopia reported the lowest level [10–12]. Several factors were associated with higher patient satisfaction, which includes the availability of drug, sign and direction indicators, social distancing measures, and hygiene facilities at healthcare centres [7]. Patient satisfaction is essential for the early detection and effective treatment of chronic diseases, which can result in improved clinical outcomes [13]. However, some studies found that the pandemic led to a decrease in patient satisfaction, especially for those with chronic diseases. Factors contributing to this decrease included the unavailability of drugs and the lack of preparedness of some healthcare facilities to handle these conditions. Overall, patients’ expectations were highest for the assurance provided by staff, followed by reliability, responsiveness, empathy, and tangibles [14]. However, there were areas that needed improvement, such as toilet and hygiene facilities and the explanation of illness and treatment by doctors [12]. The review also identified some human resource planning practices that positively influenced patient satisfaction, while others had a negative impact [15]. These findings highlight the importance of evaluating patient satisfaction during a crisis like the COVID-19 pandemic to identify areas that need improvement in healthcare systems. By addressing these issues, healthcare systems can become more patient-centered and better equipped to handle future crises.

**Fig. 3:**
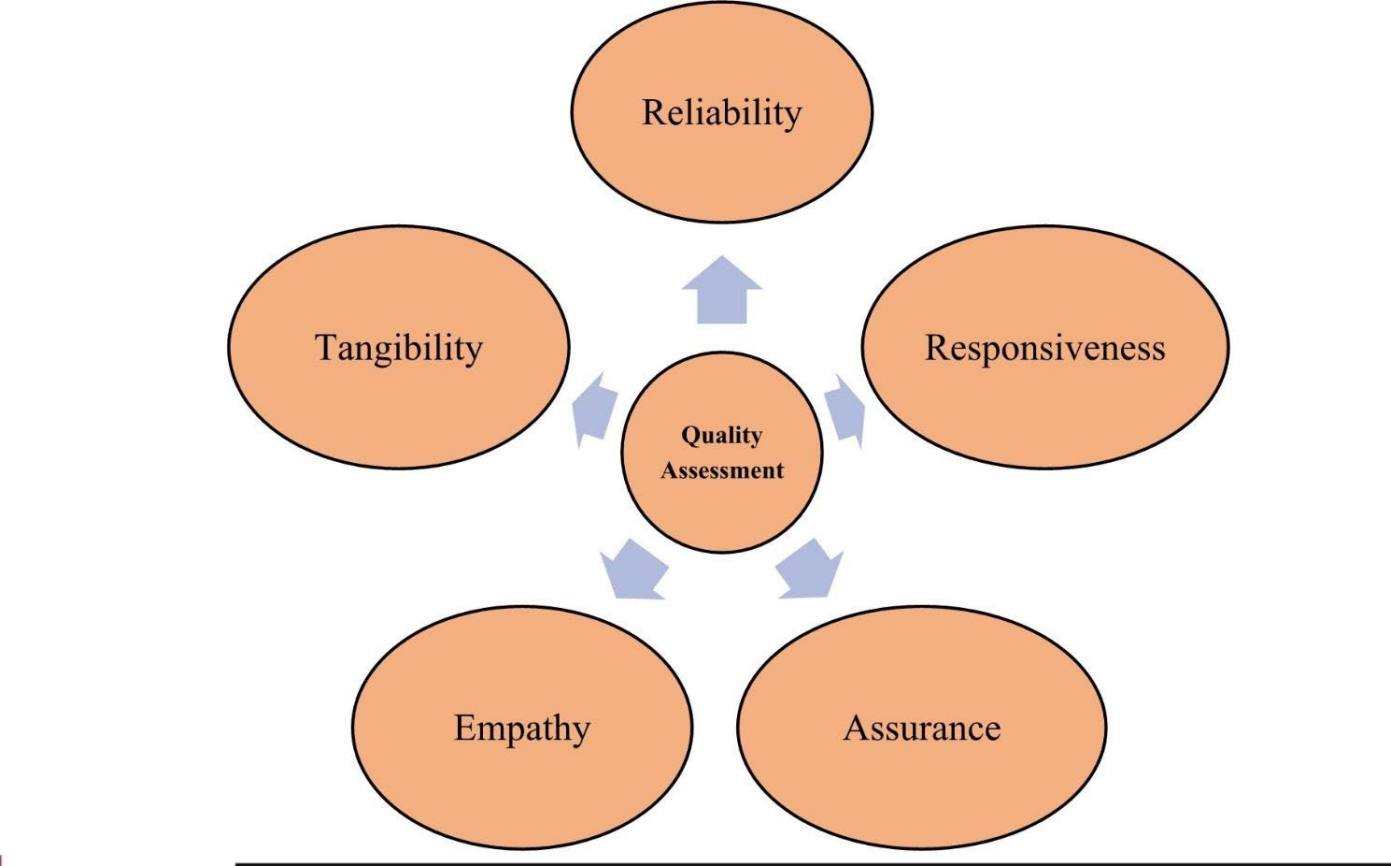
Factors for Quality Assessment

Overall, the studies suggest that healthcare providers should focus on providing high-quality care, effective communication, safety measures, and ensuring the availability of PPE to improve patient satisfaction during the COVID-19 pandemic. According to a service quality model developed by Parasuraman, Zeithaml, and Berry, there are five factors that determine quality, and they are listed in order of relevance to the patients/consumer. The five factors are Reliability, Responsiveness, Assurance, Empathy, and Tangibility (Fig. 3) [16].

Based on the above-mentioned quality determinants, this systematic review attempts to discuss the included studies for thematic evaluation based on the five factors listed above.

### Reliability

Reliability can be defined as the ability to perform the services which are promised in a reliable and accurate manner [17]. This includes the easy availability of medical facilities, easy procedure of patient admission, appropriate waiting time and easy availability of medical professionals.

The reliability aspect, which considered all the points, was found to be 3.5 on a 4- point Likert Scale and occupied the lowest score out of all five aspects [18]. It was also revealed that to receive the laboratory results, 43.8% of the study participants waited an average of 15 to 29 min [7]. Out of all the study participants, 68.9% of the patients were found to be satisfied with the reliability aspect of patient satisfaction [8]. The majority of the respondents, i.e., 91.9% stated that the working hours of the medical facilities were convenient for them, while 87.9% of them claimed that they were satisfied with the punctuality related to their medical visits. The percentage of the respondents who stated that they were able to obtain medical aid in emergency was found to be 69.7%. However, 27.3% of the study participants claimed that they faced problem while trying to book an appointment with a doctor of their own choice [13]. According to the evaluation of the dimension of reliability, the issue that received the highest rating (92.1%) was ‘hospitals keep patient data accurate and error-free’ followed by the next most highly rated issue which was that doctors do not rush through patient examinations (86.4%) [14].

### Responsiveness

Responsiveness is the willingness to help consumers and provide services quickly [17]. This includes serving patients deftly, and responding to their complaints efficiently and on time.

The responsiveness aspect was rated at a value of 3.47 [18]. Approximately two-thirds of the patients (67.2%) claimed that hospital staff made sure all their issues were resolved prior to discharge [10]. According to 52.7% of respondents, the response to patient complaints was extremely satisfactory [12]. Only 6.9% of the patients were satisfied with the responsiveness aspect of patient satisfaction [8]. The health issue addressed by 29.3% of patients was somewhat resolved, and for 48.5% of respondents, it was completely resolved [13]. Similarly for 48.5% patients in Albania responsiveness assessment was at good level with 53% [14].

### Assurance

Assurance is the knowledge and courtesy of providers and their ability to generate trust and confidence among consumers. The provision of readily available information to the patients and a clear explanation of the drugs that have been prescribed to them constitutes the assurance aspect.

With a rating of 3.51 on a 4-point Likert Scale, the assurance component held the position with the second-highest value [18]. 93% of the patients stated that the nurses and doctors answered all their queries in a clear and understandable manner while 76.8% of the patients reported that the hospital staff always explained the name and purpose of the medication being administered. Likewise, 85% of patients concurred that they comprehended the significance of taking their prescribed prescription and a comparable share (86.5%) agreed that they had an excellent grasp on how to manage their health. 64.1% of respondents reported receiving written instructions at the time of discharge [10]. 71% of the patients also stated that the staff always cared to introduce themselves to the patients prior to the start of the treatment [11]. 69.5%, 39.3% ,and 70.7% of the patients reported that the laboratory tests, x-ray/ultrasound tests and the drugs/supplies were ordered for them, respectively. 70.5% of the patients stated that the providers explained to them clearly as to how to maintain a healthy lifestyle and 84.6% of them said the providers used a language that was easier for them to understand [7]. The majority of patients (56.7%) indicated that the doctors spend between two and five minutes of their time examining patients. Approximately one-third of the patients (33.3% and 37.3%) revealed that the doctors provided an explanation regarding the illness and its treatment, respectively. Inquiries about the admission and discharge processes revealed that 58.7% and 32.7% of respondents, found it to be very satisfactory and satisfactory, respectively [12]. 19.5% of the patients were found to be satisfied with the assurance aspect of patient satisfaction [8]. Among all the criteria evaluated for assurance, the highest rating was for the security measure “Patient data is stored using the principle of confidentiality,“ with 92.8% and the alternative measure “Doctors have very good knowledge and technical skills” received a rating of 90.3% [14].

### Empathy

Empathy can be defined as a willingness to care and providing personal attention to customers [17]. This would include non-discriminatory behavior on the part of the healthcare providers towards patients, and the friendly attitude and good listening ability of the medical professionals.

The empathy aspect had the greatest value of the five factors, i.e., 3.52 [18]. 77.6% of the patients said that the nurses and doctors always treated them politely, showed them respect, and paid close attention to what they had to say. 85% of the patients who were surveyed said that staff members did take their preferences and those of their caregivers into account [10]. 94% of the patients reported that they were only rarely able to communicate with their friends and family members during their stay in the hospital [11]. The majority of respondents expressed great satisfaction with how healthcare professionals behaved. According to 64.7%, 68.7%, and 63.3% of the respondents, respectively, the actions of doctors, nurses, and paramedical staff, as well as cleaning employees, were extremely satisfactory. Most respondents (47.3%) found the overall cleanliness to be satisfactory, while 10.7% found it to be unsatisfactory to extremely unsatisfactory [12]. 64.3% of the patients were found to be satisfied with the empathy aspect of patient satisfaction [8]. The majority of patients reported that “Medical staff respects the privacy of patients,“ which received a positive evaluation of 84.9% and the alternative aspect “Staff provides patients with personal attention,“ received a positive evaluation of 81.6% [14].

### Tangibility

Tangibility is the appearance of physical facilities, equipment, personnel, and communication materials [16], which would include the availability of complete facilities for medical care and practice, cleanliness of the place of practice and the location of the practice to be easily reachable.

The tangibility aspect had a value of 3.48 [18]. 89.2% of patients claimed that the housekeeping always maintained their room and bathroom clean. Similar to this, 91.3% of patients stated that the area around their premises or surrounding their room was quiet and free of disturbances at night [10]. Three-fourths of patients claimed that there were no signs or direction indicators at the medical facilities they were using. 89% of the overall respondents confirmed about the presence of water at the medical facility. Patients were 3.26 times more likely to report being satisfied than their counterparts if they were able to use the sign and direction markers in the medical institution. Patients who received some or all the prescribed medications from the medical facility were 3.7 times more likely to report being satisfied than those who did not. At the hospital’s entrance, patients who received alcohol for hand washing were 2.7 times more likely to be satisfied than those who did not. Patients were found to be 4.4 times more pleased than their counterparts when hand sanitizer was available at the hospital entrance. Patients who obtained hand sanitizer at the health facility door were 4.5 times more likely to be satisfied than their counterparts [7]. 100% of the study participants were satisfied with the availability of a bed, lighting, and fans. Most of the patients were satisfied with the available facility. Approximately 98.7% of respondents were satisfied with the drinking water facility. According to 98% of respondents, services related to drugs and investigations were extremely satisfactory. According to the 44% of respondents, toilet facilities were adequate [12]. Unskilled or inadequately trained employees was found to be one of the leading causes of patient dissatisfaction [15]. 13.1% of the patients were found to be satisfied with the tangibility aspect of patient satisfaction [8]. Among various tangibility aspects “Hospital staff have visually clean appearance” was rated maximum with 88.1% followed by the alternatives “Rooms are quiet” and “Rooms and the bathrooms are clean,” with 64.9 and 45.8% respectively [14].

#### Limitations

This Systematic review has certain limitations. One of the main limitations is the presence of significant heterogeneity found in the various regimens such as durations, center settings, and participant characteristics across the studies included in the review; which may affect the generalizability of the findings and thus creating a roadblock in conducting the meta-analysis. Additionally, many of the studies suffer from sources of bias, which could impact the validity and reliability of the results but still had to be included due to the limited number of studies available that met the inclusion criteria. Inclusion of fewer studies also led to a skewed effect while performing thematic evaluation.

## Conclusion

This systematic review concluded that patient satisfaction level with respect to the quality of health care provided was highest in Saudi Arabia (98.1%) followed by India (Madhya Pradesh) (90.6%) and U.K. (90%). We have discussed Patient satisfaction levels across five different aspects: - Reliability, Responsiveness, Assurance, Empathy, and Tangibility. It was found that the aspect of empathy had the greatest value of the five factors, i.e., 3.52 followed by Assurance with a value of 3.51. Given the multifactorial nature of different aspects influencing patient satisfaction with the quality of health care, further research is needed to identify additional factors especially from the patient’s perspective and explore context-specific strategies in order to increase the quality of health care. Thus, it can be concluded that a patient satisfaction survey is an effective, affordable, reliable, and quick method to assess the quality of health care and should be conducted periodically in order to evaluate and monitor ongoing health care delivery system. In light of this review, future research focusing on identifying factors that contribute to high patient satisfaction and positive outcomes in healthcare facilities during the pandemic is the need of the hour. This will aid in exploring whether these factors are consistent across different countries and healthcare systems globally. Additionally, incorporating comparison groups and non-self-reported data in future studies could provide more robust evidence regarding the effects of the pandemic on patient satisfaction and outcomes.

## Data Availability

The data set analysed during the current study are available in PubMed **(**https://pubmed.ncbi.nlm.nih.gov/), Google Scholar (https://scholar.google.com/) and Embase (https://www.elsevier.com/en-in/solutions/embase-biomedical-research).
